# Efficacy and Methodology of Remote Heart Rate Variability Biofeedback Interventions for Mental Health: A Systematic Review and Meta-Analysis

**DOI:** 10.1007/s10484-025-09750-w

**Published:** 2025-11-27

**Authors:** Sophia Vann-Adibe, Harry Kam Hung Tsui, Hui Quan Zhou, Zhuoma Ciren, Chao Li, Sherry Kit Wa Cha

**Affiliations:** 1 Department of Psychiatry, School of Clinical Medicine, LKS Faculty of Medicine, The University of Hong Kong, Hong Kong, China; 2 Department of Psychiatry, Queen Mary Hospital, Room 219, New Clinical Building, 102 Pokfulam Road, Hong Kong, Hong Kong SAR China

**Keywords:** Heart rate variability, Biofeedback, Mental health

## Abstract

Heart rate variability biofeedback (HRV-B) is a technique that has been shown to have benefits for both physical and mental health conditions. The aim of the present systematic review and meta-analysis is to describe the methodological approach and examine the efficacy of remote HRV-B interventions for mental health symptoms, including depression, stress, anxiety, and sleep as well as heart rate variability (HRV), a measure of autonomic function. A systematic literature search was conducted from five databases and eighteen studies with a total of 1352 subjects from different populations were included in this systematic review and meta-analysis based on the inclusion criteria. Meta-analysis showed a medium effect size of HRV-B for improving both depression (*g* = − 0.41 [− 0.049, − 0.772], *p* = 0.026, I^2^ = 72.623%, k = 10) and HRV (*g* = 0.443, [0.718, 0.167], *p* = 0.002, I^2^ = 56.81%, k = 10) compared to control conditions. These results remained significant after sensitivity analyses excluding high-risk, non-randomized, and outlier studies. Effect for stress was not significant (*p* = 0.152, k = 8). Meta-regressions identified study and protocol characteristics as significant moderators of the effect on HRV, stress, and anxiety. Specifically, maximizing resonance, screen on the device, less lab visits, less than twenty minutes of practice time per day, and female gender were beneficial for the intervention effectiveness. Overall, this study indicates that HRV-B is an effective intervention for decreasing depressive symptoms and increasing HRV across populations, and some intervention variables may influence the intervention outcomes.

**Supplementary Information:**

The online version contains supplementary material available at https://doi.org/10.1007/s10484-025-09750-w.

## Introduction

### Heart Rate Variability and Mental Health

The Autonomic Nervous System is a part of the Peripheral Nervous System that controls our involuntary response to stimuli and is divided into the Sympathetic (SNS) and Parasympathetic (PNS) Nervous System. The SNS is sometimes considered the “fight or flight” system and responds during stressful situations to ready the system for action which includes increasing heart rate. The PNS is also known as the “rest and digest” system and is responsible for returning the system to equilibrium and decreasing heart rate (Karemaker, 2017). Heart rate is therefore a measure of the current state of the autonomic nervous system, and heart rate variability (HRV) is a measure that represents how well the system is able to adapt to a changing environment (Pham et al., 2021). HRV is the change in time intervals between consecutive heart beats.

A higher HRV indicates a greater resilience to stress as it demonstrates that the system can be brought to equilibrium faster. There is evidence that HRV is reduced in individuals with various psychiatric conditions, including depression, PTSD and psychosis (Jung et al., 2019; Ramesh et al., 2023). Psychiatric medication has also been found to decrease HRV, however unmedicated patients still have reduced HRV (Alveres et al., 2015). This indicates that there is likely altered autonomic function in a wide range of psychiatric conditions and emphasizes the need for effective interventions to restore autonomic function in these conditions.

### The Effect of Heart Rate Variability Biofeedback on Mental Health

There is evidence that voluntary slow breathing can increase HRV, both in the short-term and in the long-term (Laborde et al., 2022). The mechanism by which it does this is posited to be through the activation of baroreceptors, mechanical sensors that decrease heart rate when blood pressure increases. As arterial pressure decreases during inhalation and increases during exhalation, arterial baroreceptors are activated during slow breathing. This is known as the baroreflex, and a stronger baroreflex is associated with higher HRV (Pham et al., 2021). Heart Rate Variability Biofeedback (HRV-B) is an intervention approach that uses real-time representation of physiological data, such as heart rate and HRV, to help participants maximize their HRV while breathing. HRV-B has been shown to increase baseline HRV through various mechanisms including the baroreflex (Lehrer & Gevirtz, 2014).

It has been found that HRV-B can improve depression, anxiety, and emotional and physical health. A meta-analysis found a medium effect size for HRV-B on reducing depressive symptoms across multiple populations (Pizzoli et al., 2021). In a systematic review and meta-analysis, small to medium effect sizes for the effect of HRV-B on anxiety, depression, anger, and athletic/artistic performance were demonstrated (Lehrer et al., 2020). Similarly in another earlier review of a transdiagnostic population, a large effect of HRV-B on anxiety was found (Goessl et al., 2017). However, evidence for the effects of HRV-B on stress is more mixed. To our knowledge, no reviews have been conducted on the specific influence of HRV-B on perceived stress. While some empirical studies (Brinkmann et al., 2020; Ribeiro et al., 2023) found that HRV-B significantly reduced perceived level of stress in various populations, others did not find a significant impact of HRV-B on perceived stress (Lehrer et al., 2020; Solarikova et al., 2021).

### Research Gaps

To our knowledge, no reviews have examined quantitatively the effect of varied intervention protocol on intervention outcomes in hybrid or remote studies. A relatively recent systematic review on HRV-B described qualitatively different HRV-B methods (Lalanza et al., 2023) however didn’t examine their quantitative effects on intervention outcomes. There have also since been empirical studies that use methods that employ recent technology such as VR devices (Cuneo et al., 2023) and smartphone apps (Vacher et al., 2023). These studies often used remote or hybrid approaches which vary from in person interventions in their methodology, however this approach has not been examined specifically in previous reviews. Other systematic reviews and meta-analyses examined the effect of HRV-B on various psychological and physiological outcomes and examined some study design factors such as intervention duration and number of lab visits effects (Lehrer et al. 2020) however did not quantify the effect of practice time, HRV-B device and HRV-B method on intervention effectiveness. Similarly, a meta-analysis which focused on the effect of HRV-B on depressive symptoms (Pizzoli et al. 2021) examined duration of intervention, year of publication and questionnaire used however did not investigate the impact of other study variables on the intervention outcomes. Additionally, evidence for the effect of HRV-B on perceived stress is still inconclusive. Remote HRV-B interventions are also growing in popularity (Birk et al., 2024; Economides et al., 2020) however no reviews have described the methods of remote HRV-B interventions in particular. Evidence from individual studies indicate the method is feasible and acceptable, however more research is needed to determine the overall efficacy of remote HRV-B interventions and the impact of study variables on the results.

### Aim of the Review

The aim of the present systematic review and meta-analysis is to examine how study protocol affects the outcomes of remote or hybrid HRV-B interventions for different symptoms across populations. No restrictions on population are applied to increase the generalizability of our results and ensure sufficient sample size. We will also summarize the study protocols and highlight some important study variables based on the results of our analysis. Results of this review can provide evidence-based guidance on HRV-B intervention protocols for different psychological outcomes.

## Methods

### Search and Screening Strategy

A systematic search of the literature was conducted on September 27th, 2024 in the following electronic databases: PubMed, PsycInfo, Cochrane Central Register of Controlled Trials (CENTRAL), Web of Science, and MEDLINE. The keywords used were the following: (“Heart Rate Variability Biofeedback” OR “HRV Biofeedback” OR “HRVB” OR “HRV-B” OR “Respiratory Sinus Arrhythmia Biofeedback” OR “RSA Biofeedback”) AND (“Psychiatric” OR “Mental Health” OR “Stress” OR “Depression” OR “Psychological” OR “Anxiety” OR “Panic” OR “Post Traumatic Stress Disorder” OR “PTSD” OR “Substance Use” OR “Psychosis”) AND (“Intervention” OR “Treat*”).

The articles identified from the database search were imported into the Covidence software for duplication removal and screening. Covidence automatically removed duplicate articles, after which we did a manual screening of title and abstract followed by full text review. Studies that were (1) published in English; (2) human intervention studies; (3) examined any mental health symptom as an outcome with HRV as a secondary outcome; (4) had treatment as usual or waitlist control conditions; (5) used empirical quantitative analysis were included. No restrictions were placed on age of participants or study date. The exclusion criteria included (1) fully in person or single session interventions; (2) studies that did not use biofeedback during home breathing sessions; (3) other types of papers such as theses, conference abstracts, and case reports; (4) replicated samples; (5) those that did not respond to requests to provide sufficient data to allow for meta-analysis.

**Fig. 1:**
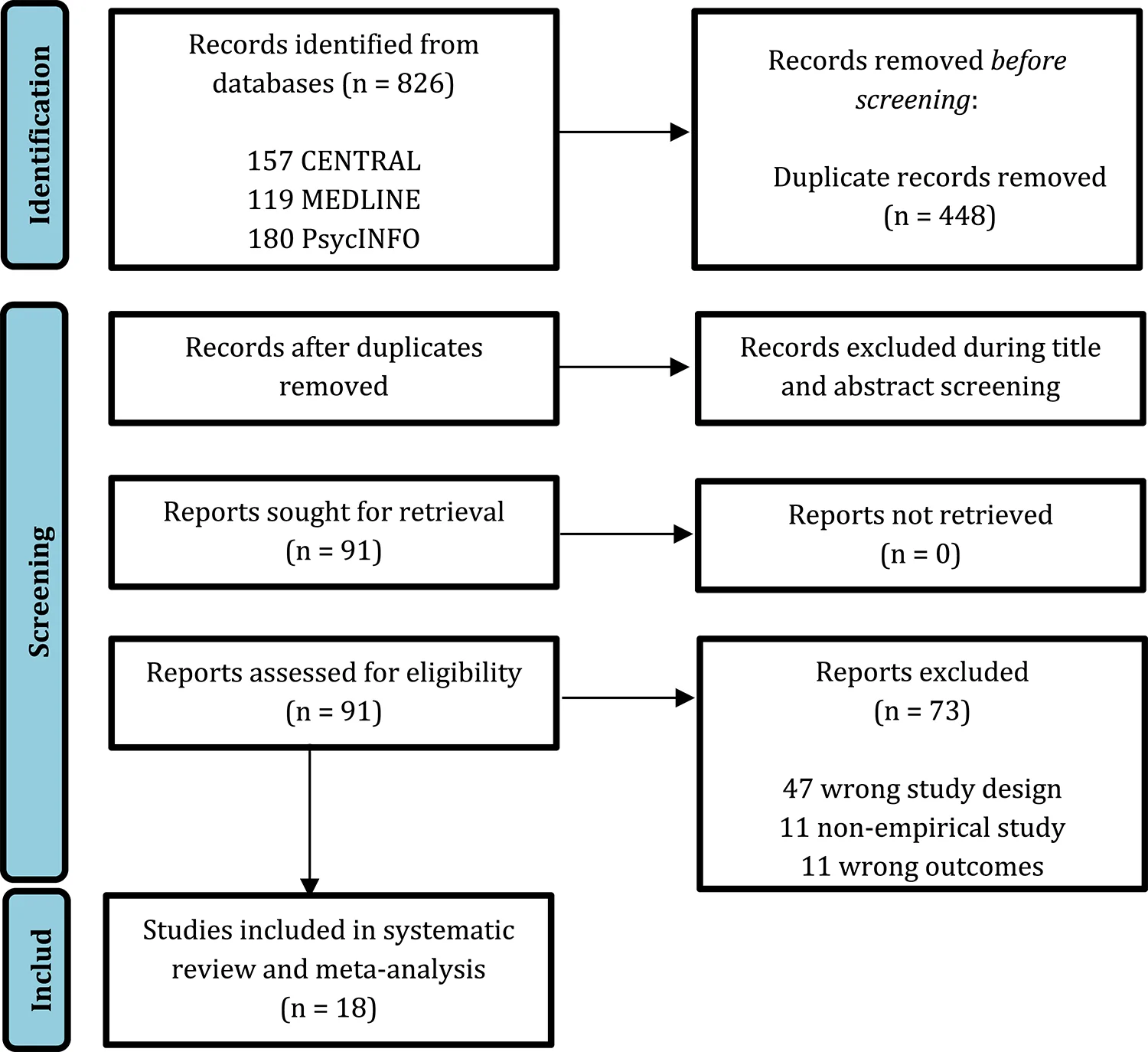
Preferred Reporting Items for Systematic Reviews and Meta-Analyses (PRISMA) flow diagram showing study selection

Screening and data extraction was performed by two independent reviewers (SVA and KHT) with any discrepancies resolved through discussion or consultation with a third reviewer (KWC). Extracted data was stored in a secure database for data management. Preferred Reporting Items for Systematic Reviews and Meta-Analyses (PRISMA) guidelines were followed during the study selection process. The flowchart is shown in Fig. 1.

### Risk of Bias Assessment

Risk of bias was assessed with the Cochrane risk-of-bias tool version 2. Included studies were assessed for bias due to the randomization process, bias due to deviations from the intended intervention, bias due to missing data, bias due to outcome measurement, and bias due to selective reporting of results. The studies were rated as “low,” “some concerns” or “high” risk of bias based on the Cochrane guidelines.

### Data Extraction, Synthesis and Analysis

We first provide a narrative synthesis to integrate and interpret the methods and results of the included studies by examining the typical study protocol and describing it qualitatively. This includes describing the HRV-B method, HRV-B device, and structure of a typical intervention.

For the meta-analysis, the key outcomes were psychometric symptoms and HRV. The sample size, means and standard deviations of psychiatric symptom outcomes and the Standard Deviation of Normal to Normal intervals (SDNN) measure of HRV results pre (T1) and post (T2) intervention for experimental and control groups were extracted from each study. Additionally, other relevant factors include age, gender, choice of HRV-B device, HRV-B method, study duration, practice time, and number of lab visits were extracted to perform metaregression. Meta-analysis for each outcome was performed if there were at least 3 studies that included the outcome. The effect size used was *Hedges’ G*, a measure of standardized mean difference that considers sampling variance differences between groups. Effect sizes for each study were computed using the *metafor* package. A random effects model for each outcome was calculated using the restricted maximum likelihood method. Heterogeneity among the effect sizes was assessed using Higgin’s I^2^, which measures the percentage of variability in the true effect size estimation that is due to heterogeneity rather than chance.

Univariable meta-regressions were performed using study protocol and characteristics as the moderator. Categorical meta-regressions were performed if each category had at least 2 studies. For practice time a cutoff of twenty minutes was used to separate the studies into two categories as it was the closest to the average practice time. Subgroup meta-analyses were conducted to examine the effect of population (physical or psychological condition) on intervention results if each category had at least 2 studies. For stress categorical meta-regression analyses, the mean perceived stress scale (PSS) score at baseline was used as a cutoff to indicate more stressed subjects. Baseline stress was also used as a continuous variable for meta-regression. Sensitivity analyses were conducted to test the robustness of the results. This included leave-one-out analyses to determine the effect of each study on the random effects results in addition to excluding high-risk and non-randomized studies, as well as outliers, and recalculating the random effects. Additionally, relative risk of drop out for the experimental group in comparison to the control group was calculated for each outcome and the Fisher Exact Test was used to calculate the *p*-value as dropouts were sometimes under 5. The threshold of significance was set at < 0.05 for all analysis. All analysis was conducted using R Studio (R 4.4.1).

## Results

### Literature Search

A total of 826 records were identified from the database search (Fig. 1). After the first screening of title and abstract, 91 studies remained, of which 73 were excluded due to not meeting the inclusion criteria. The remaining 18 studies were included in the present systematic review and meta-analysis. Among which, 8 studies included individuals with psychiatric conditions, 8 studies included individuals with physical conditions, one with healthy subjects and one with athletes.

### Description of Common Protocol for HRV-B

**Fig. 2:**
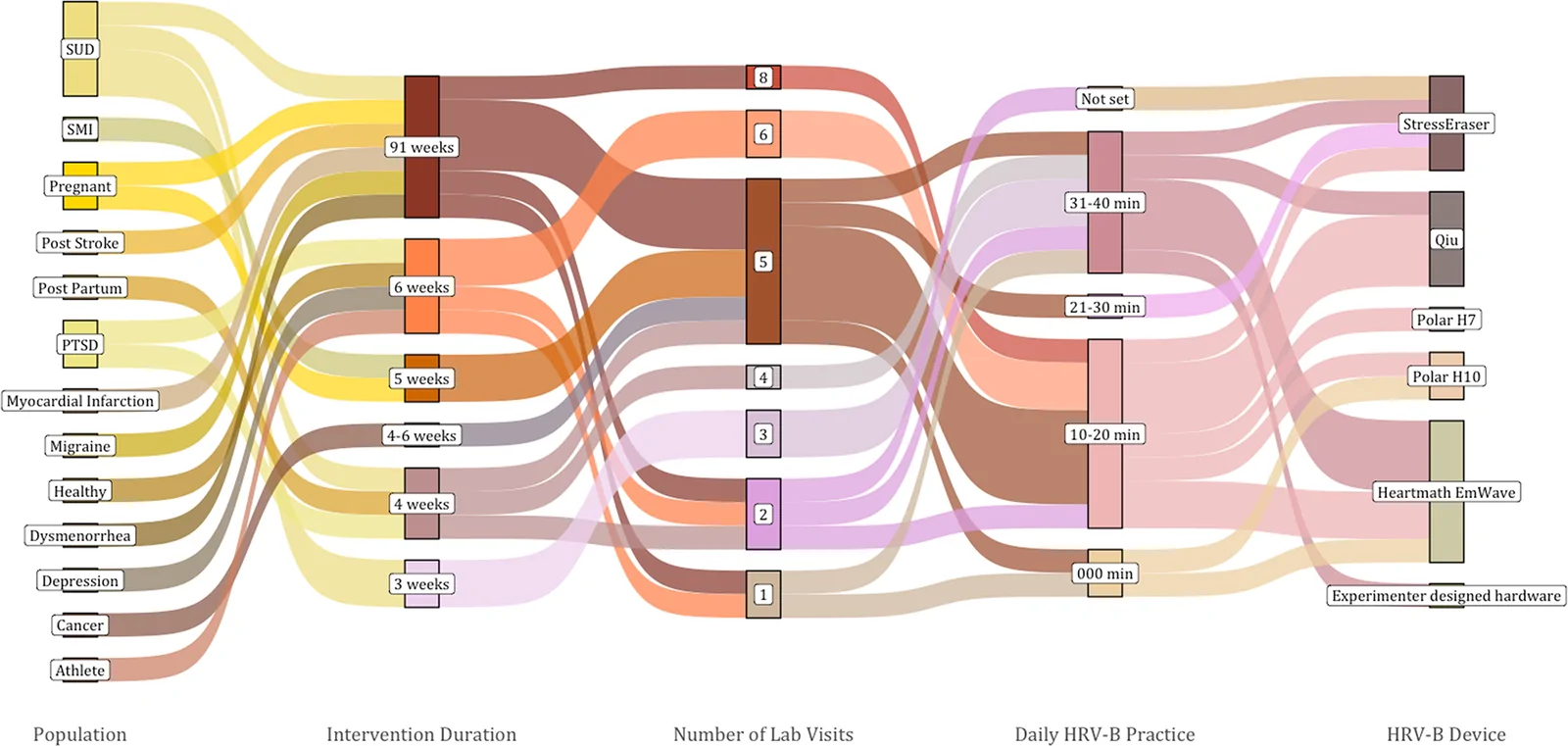
Sankey plot of study and protocol characteristics

The characteristics of the selected studies are summarized in Table 1 and the key intervention and protocol characteristics can be visualized in the Sankey diagram shown in Fig. 2. Based on the eighteen selected studies of remote HRV-B interventions, the average intervention length was 7.2 weeks (SD 3.6), with 4.1 lab visits (SD 1.9) and an average practice time of 22.1 min per day (SD 11.2). The most used biofeedback device was the HeartMath emWave (5 studies) followed by the StressEraser (4 studies) and Qiu (4 studies). The majority of interfaces were integrated into the device (13 studies) while the other studies used apps or VR software. The average dropout rate for the experimental group was 17.05% (SD 12.9) while the rate for the treatment as usual or waitlist control group was 8.3% (SD 9.5).

**Table 1:** Summary table of study characteristics and protocol

References	Study design	Control	Country of study	Population	Average age (SD)	Sample size (num Female)	Intervention duration	Number of lab visits	HRV-B practice	Outcome measurement (psychometric)	Method	Devices and interface
Brinkmann et al. (2020)	RCT	WL	Germany	Healthy	43.27 (10.45)	47 (26)	6 weeks	1	30 min daily	BDI-II, FFA-14	MR	Qiu, SoD
Burch et al. (2020)	RCPS	WL	USA	Cancer	59.5 (2.55)	34 (29)	4–6 weeks	5	15 min daily	BDI-II, PSS	MR	Heartmath emWave, SoD
Cui et al. (2020)	RCT	TAU	China	Pregnant	28.7 (3.52)	20 (20)	12 weeks	2	30 min daily	PSQI	MR	Experimenter designed hardware and app
Cuneo et al. (2023)	RCPS	WL	USA	Migraine	42.38 (15.21)	36 (30)	12 weeks	1	10 min 3 × per week	PHQ-8, PSS	SM	Polar H10, Oculus VR software
Eddie et al. (2014)	NRPS	TAU	USA	SUD	21.85 (1.85)	41 (0)	3 weeks	3	20 min 2 × daily	PACS, PSS	SM	Heartmath emWave, SoD
Eddie et al. (2018)	NRCT	WL	USA	SUD	23.6 (5)	36 (10)	12 weeks	5	15 min 2 × daily	PSS, PACS, BAI, BDI-II	SM	StressEraser, SoD
Kim (2014	QEP	TAU	USA	SUD	21.8 (1.85)	42 (0)	3 weeks	3	20 min 2 × daily	PSS, PACS *	SM	Heartmath emWave, SoD
Kudo et al. (2014)	QEP	TAU	Japan	Post-Partum	32.1 (6.21)	55 (55)	4 weeks	2	Daily, no set duration	EPDS	MR	StressEraser, SoD
Limmer et al. (2022)	RCT**	TAU	Germany	Myocardial Infarction	60.5 (9.37)	46 (7)	12 weeks	5	5 min 3 × daily	No relevant assessments	MR	Qiu, SoD
Schumann et al. (2022)	RCPS	WL	USA	PTSD	50.6 (14.38)	35 (5)	4 weeks	5	5 min daily	PCL-5, BDI-II	SM	Heartmath Inner Balance Device, Inner Balance app
Schumann et al. (2022)	NRPS	WL	Germany	Depression	41 (15)	25 (19)	6 weeks	2	2 × 11 min, 5 × per week	BDI, STAI, PSS	SM	Polar H10, Smartphone app
Tatschl et al. (2020)	RCPS	TAU	Austria	Severe Mental Illness	48.7 (9.4)	68 (43)	5 weeks	5	10 min 2 × daily	BDI-II	MR	Qiu, SoD
Vacher et al. (2023)	RCPS	NT	Switzerland	Athlete	15.45 (2.33)	27 (11)	6 weeks	6	10 min 2 × daily	PSS	SM	Polar H7, Breath/Breath + app
Vagedes et al. (2019)	RCT	TAU	Germany	Dysmenorrhea	29.7 (8.0)	40 (40)	12 weeks	5	15 min daily	SF-12	MR	Qiu, SoD
van der Zwan et al. (2019)	RCT	WL	the Netherlands	Pregnant	31.6 (5.9)	50 (50)	5 weeks	5	10–40 min daily, increasing	DASS (D, A, S), PSQI	SM	StressEraser, SoD
Wang et al. (2024)	QEP	TAU	Taiwan	Post Stroke	60.1 (12.3)	62 (19)	12 weeks	8	10 min 2 × daily	PSS	PP	StressEraser, SoD
Winstead (2019)	RCPS	TAU	USA	PTSD	54 (11)	85 (28)	6 weeks	6	15 min daily	BDI-II, PSS	MR	Heartmath emWave, SoD
Yen et al. (2022)	RCT	TAU	Taiwan	SUD	37.27 (9.53)	61 (0)	4 weeks	4	20 min 2 × daily	PSQI, CES-D,	SM	Heartmath emWave, SoD

RCPS, Randomized Controlled Pilot Study; NRPS, Non-Randomized Pilot Study; RCT, Randomized Controlled Trial; NRCT, Non-Randomized Controlled Trial; RPS, Randomized Pilot Study; QES, Quasi-Experimental Study; WL, Wait List Controlled; TAU, Treatment As Usual; NT, No Treatment; SUD, Substance Use Disorder; PTSD, Post-Traumatic Stress Disorder; PCL-5, PTSD Checklist-5; BDI-II, Beck Depression Index-II; PACS, Penn Alcohol Craving Scale; PSS, Perceived Stress Scale; PSQI, Pittsburgh Sleep Quality Index; BAI, Beck Anxiety Index; DASS-21, Depression Anxiety Stress Scales; FFA-14, Frierburg Mindfulness Inventory; STAI, State-Trait Anxiety Inventory); CES-D, Center for Epidemiological Studies Depression Scale; SF-12, Short Form Health Survey; PHQ-8/9, Patient Health Questionnaire. MR, Maximize Resonance; SM, Stepped Method; PP, Preset Pace. SoD, screen on device. * No post intervention data. **RCT not registered

Despite a varied intervention protocol of HRV-B across studies, the overall intervention procedures were similar (S Fig. 1). It generally involved an initial visit to train the participant in the HRV-B method and determine resonance frequency if the study used that method. Otherwise, participants would breathe at a preset pace or try to maximize their HRV resonance in real time. Pre-intervention assessments would also be taken at this time. Then the participants would undergo their first session of HRV-B under the guidance of the biofeedback facilitators. The device was generally a stand-alone device with an integrated screen that served as the HRV-B interface. Participants would then take the device home with instructions on how to practice daily with multiple lab visits throughout the intervention for most studies. During the final visit, participants would return the device and take the post intervention assessments and measurements.

### Types of HRV-B Intervention

Based on the different protocols of the included studies, the methods for determining breathing pace during HRV-B were divided into three categories: stepped method (SM: k = 9 studies), maximize resonance (MR: k = 8 studies), and preset pace (PP: k = 1 study). The stepped method follows the protocol proposed previously (Lehrer et al., 2000) and involves an assessment prior to the start of the intervention to determine the participants individual resonance frequency. The assessment consists of having the individual breathe at various paces for two minutes each while measuring HRV, usually starting at 6.5 breaths per minute (b/m) and decreasing by half a minute until 4.5 b/m. The breathing pace that generates the maximum HRV is taken to be the resonance frequency (RF) and used for the duration of the intervention. The maximize resonance method doesn’t prescribe a specific breathing pace but rather guides participants to breath in a way that maximizes their HRV or cardio-respiratory coupling in real-time following cues such as a green/red light or the waveforms of their HRV and respiration. The final method involves a preset breathing pace, which is usually 6 b/m. This method is usually employed when the target population has physical or cognitive limitations to improve compliance (Wang et al., 2024).

### The Effect of HRV-B on Psychological Outcomes and HRV

**Fig. 3:**
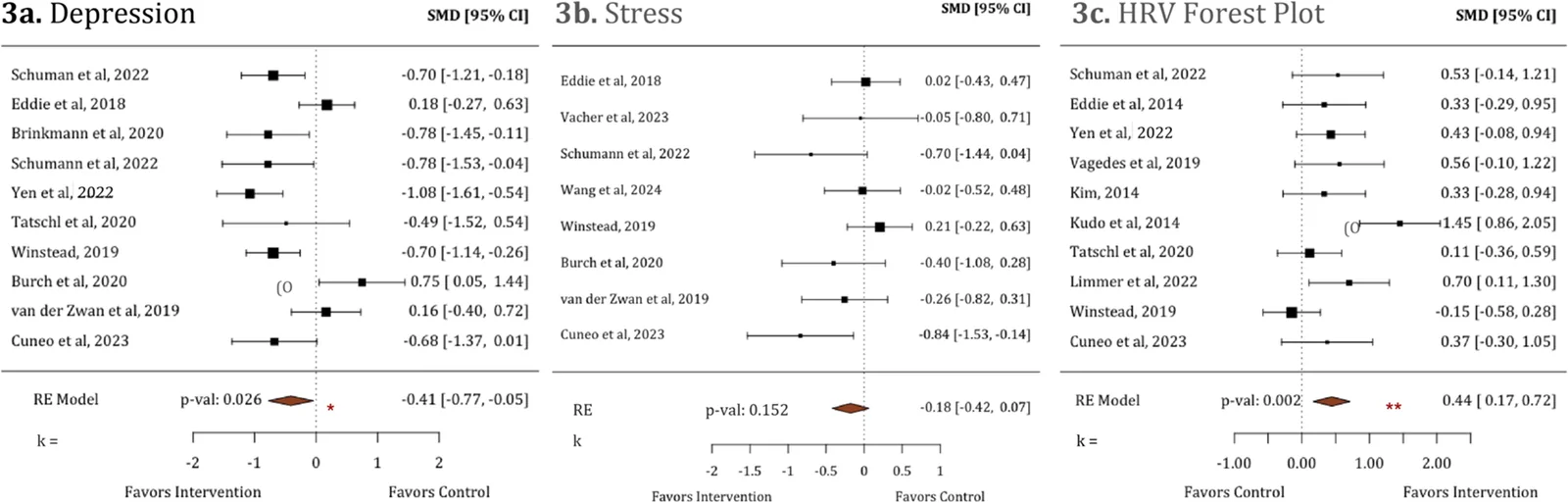
Forest plot of *Hedge’s g*

Meta-analysis showed a medium effect size for both depression (*g* = − 0.41, [95% CI − 0.049, − 0.772], *p* = 0.026, I^2^ = 72.623%, k = 10) and HRV (*g* = 0.443, [95% CI 0.718, 0.167], *p* = 0.002, I^2^ = 56.81%, k = 10) compared to control conditions (Fig. 3). These results remained significant after sensitivity analyses excluding high-risk, non-randomized, and outlier studies (S Table 1).

The effect for stress was not significant (S Table 1) nor was the analysis for anxiety (S Table 1). Meta-analysis for sleep showed a large effect size (g = − 0.753, [95% CI − 0.092, − 1.413], *p* = 0.026, I^2^ = 67.14%), however only three studies reported sleep as an outcome and results were non-significant after excluding the high risk studies.

Leave one out sensitivity analyses (S Fig. 3) remained significant for depression for 6/10 studies, was significant for stress for 1/8 studies, and was significant for HRV for 8/10 studies. For sleep, 2/4 studies were significant and for anxiety 1/4 studies was significant.

### The Influence of Study and Protocol Variables on Intervention Outcomes

Meta-regressions with gender, study and intervention protocol characteristics as moderators were mostly nonsignificant except for two variables for stress (Table 2), including number of lab visits (k = 8, QM = 6.346, QMp = 0.012, β = 0.134, SE = 0.053) and gender (percent female) (k = 8, QM = 5.885, QMp = 0.015, β = − 0.883, SE = 0.364). Tests of moderators for depression and HRV were nonsignificant for all tested variables.

**Table 2:** Meta-regression analyses

Categorical variable	QM	QMp	k	β	SE	*p*-val
*Depression*						
Practice time	4.475	0.107				
Interface	6.721	0.081				
HRVB-method	4.572	0.102				
*Stress*						
Practice time	2.137	0.343				
Interface*	6.542	**0.038**				
Screen on device		5	0.027	0.115	0.815	
Smartphone app/other software*		3	0.547	0.215	**0.011**	
HRVB-method	3.115	0.374				
*HRV*						
Practice time*	8.291	**0.016**				
20 min or less*		6	0.279	0.132	**0.035**	
Over 20 min		3	0.370	0.189	0.050	
Interface*	9.122	**0.010**				
Screen on device**			8	0.443	0.162	**0.006**
Smartphone app/other software			2	0.454	0.352	0.197
HRV-B method*	9.101	**0.011**				
Maximize resonance*			5	0.488	0.205	**0.017**
Stepped method			5	0.399	0.215	0.064

Continuous variable	k	QM	QMp	β	SE
*Depression*					
Study duration	10	0.290	0.590		
Total practice time	10	0.545	0.460		
Number of lab visits	10	1.445	0.229		
Practice time per day	10	0.014	0.906		
Age of participants	10	0.029	0.865		
Gender(% female)	10	1.990	0.158		
*Stress*					
Study duration	8	0.000	0.990		
Total practice Time	8	1.313	0.252		
Number of lab visits*	8	6.346	**0.012**	0.134	0.053
Practice time per day	8	1.967	0.161		
Age of participants	8	0.003	0.957		
Gender(% female)*	8	5.885	**0.015**	− 0.883	0.364
*HRV*					
Study duration	10	0.017	0.897		
Total practice time	9	2.23	0.135		
Number of lab visits	10	1.527	0.217		
Practice time per day	9	0.004	0.947		
Age of participants	10	0.543	0.461		
Gender(%Female)	10	0.645	0.422		

Negative values for psychometric outcomes (Depression and Stress) indicate greater improvement, positive values for HRV indicate greater increase. Bold indicates significance level < 0.05.

k, number of studies; QM, omnibus test of moderators; QMp, *p*-value of QM; β, slope; SE, standard error. *< 0.05 significance level; **< 0.01 significance level

For categorical meta-regressions (Table 2), the test of moderators was nonsignificant for the depression outcome. For stress, choice of interface was significant (QM = 0.038, QMp = 0.038) with smartphone app/other software usage being the significant moderator (k = 3, β = 0.547, SE = 0.215, *p*-val = 0.011). For HRV, the significant moderators included practice time (QM = 8.291, QMp = 0.016), interface (QM = 9.122, QMp = 0.01), and HRV-B method (QM = 9.101, QMp = 0.011). For practice time, 20 min or less was the significant moderator (k = 6, β = 0.279, SE = 0.132, *p*-val = 0.035). For interface, the significant moderator was whether the screen was on the device (k = 8, β = 0.443, SE = 0.162, *p*-val = 0.006). For the HRV-B method the significant moderator was the use of the maximize resonance method (k = 5, β = 0.488, SE = 0.205, *p*-val = 0.017).

Population subgroup meta-analyses were conducted to see the effect of population type on outcome (S Table 2). For depression outcomes, populations with a psychological condition had significant improvement with intervention (*g* = − 0.583, [95% CI − 0.192, − 0.974], *p* = 0.003, I^2^ = 64.269%, k = 6) while for HRV outcomes, both populations with physical conditions (*g* = 0.558, [95% CI 0.928, 0.188], *p* = 0.003, I^2^ = 0%, k = 3) and populations with psychological conditions (*g* = 0.411, [95% CI 0.786, 0.036], *p* = 0.032, I^2^ = 69.554%, k = 7) had significant results. There were no significant results for the stress outcome. For diagnosis subgroup meta-regressions (S Table 3), there were no significant differences between subgroups for depression and sleep outcomes. Other outcomes were not examined as they had less than two groups for all diagnoses.

To examine whether there were differences in outcomes for those who were more stressed at baseline, a subgroup meta-regression analysis was performed for those more stressed at baseline. However, there were no significant differences between groups (S Table 4: QM = 2.875, QMp = 0.237), although the studies with moderately stressed population at baseline approached significance (β = 0.325, SE = 0.196, *p* = 0.098). We also performed a meta-regression with baseline stress as a continuous variable however there were no significant results (S Table 4: QMp = 0.445).

### Relative Risk of Dropout for Experimental Versus Control Group

The relative risk of drop out for the experimental group compared to the control group for studies with depression outcomes was 1.324 (k = 10, 95% CI [1.06, 1.586], *p*-val = 0.004), stress 1.345 (k = 8, 95% CI [1.091, 1.659], *p*-val = 0.011), HRV 1.236 (k = 10, 95% CI [1.007, 1.519], *p*-val = 0.041), sleep 1.785, (k = 3, 95% CI [1.360, 2.343], *p*-val = 0.019), and anxiety 1.681 (k = 3, 95% CI [1.301, 2.173], *p*-val = 0.004) (S Table 5).

### Risk of Bias and Publication Bias

The Egger’s tests for depression, HRV and sleep were not significant (*p* = 0.981, *p* = 0.073, *p* = 0.654) suggesting there was not significant publication bias (Supplementary Fig. 5a, c, e). The Egger’s tests for stress and anxiety were significant (*p* = 0.01, *p* = 0.029) indicating that there may be publication bias for these variables (Supplementary Fig. 5b, d).

## Discussion

### Significance of Results

In this systematic review and meta-analysis, 18 studies with a total of 1352 subjects were included. To our knowledge, it is the first meta-analysis to describe and examine in detail how various HRV-B study and intervention variables impact the results of a range of outcomes. We found that HRV-B is effective for reducing depression symptoms and increasing HRV, but not for perceived stress. HRV-B also had a significant effect on sleep, however only three studies examined this outcome and results became insignificant after excluding high risk of bias studies. There was no significant effect of HRV-B on anxiety however only three studies examined this outcome and two of them were nonrandomized and had a high risk of bias. Some important study variables that influenced study outcomes were the HRV-B method, HRV-B interface, gender, number of lab visits and practice time per day. Specifically, maximizing resonance, the screen being on the device, and less than 20 min of daily practice time were beneficial for improving HRV outcomes. Female population and having less laboratory visits during the HRV-B intervention was associated with more improvement of perceived stress.

Overall, the results of the random effects analysis show a significant medium effect size for reducing depression and for increasing HRV, suggesting that HRV-B is a moderately effective intervention for reducing depression and improving HRV. This result is consistent with the results of previous reviews (Lehrer et al., 2020; Pizzoli et al., 2021) which found that HRV-B interventions yielded small to medium effect sizes for improving depressive symptoms. When examining studies by separating the populations into those with psychological conditions and those with physical conditions, we found that significant effects of HRV-B persisted for HRV improvement in both study populations. Effects on depression persisted for populations with psychological conditions only. A smaller number of studies with depression as outcomes in populations with physical conditions might be one explanation. It might also be possible that populations with physical conditions have lower depression scores at baseline.

The lack of significant effect for stress in our study is in contrast to some previous empirical studies (Brinkmann et al., 2020; Ribeiro et al., 2023) but was in line with some other studies (Lehrer et al., 2020; Solarikova et al., 2021). One of the possible reasons for the negative finding in this study could be the high standard deviation (11.76) of baseline PSS scores in our included studies. A previous study has suggested individuals with high baseline PSS scores are more likely to show improvement with the intervention (Ribeiro et al., 2023). Though our sensitivity analysis splitting the studies into those with low and moderate baseline PSS scores did not show statistically significant results for both groups, a trend towards significance was seen in studies with moderate baseline PSS scores. Furthermore, separate meta-analyses of people with psychological conditions and those with physical condition showed a trend towards significant effect on reduction of stress in people with physical condition. These results suggested that HRV-B might have an effect on reduction of stress among a specific group of population. Further studies will be needed to understand more of the differential effect of HRV-B on stress reduction in different populations. Though there was a significantly large effect size for improving sleep, only three studies examined sleep as an outcome, and the results were no longer significant after excluding high-risk studies. There were only three studies examining the effect of HRV-B on anxiety and results were non-significant. More studies will be needed to examine the effect HRV-B on these outcomes.

Our meta-regression analyses of continuous variables found a significant but small effect of the number of laboratory visits on results of stress reduction, with more visits decreasing the effectiveness of the intervention. This result is in contrast to the finding of a previous review (Lehrer et al., 2020) that intensity of the intervention as measured by the number of lab visits did not influence intervention effectiveness. However, they did not stratify by outcome, and this may have a particular impact on stress. For example, more frequent visits could subtly increase patient stress and anxiety, as a poll indicated that 48% of Americans feel anxious before a doctor’s appointment (Patient Point, 2023). Results also suggested female gender was associated with more improvement of perceived stress. This indicates that the intervention is likely to be more effective for reducing perceived stress in women. A review suggests that women have greater parasympathetic influence on heart rate than men (Kajantie & Philips, 2006), possibly explaining why HRV-B, which mainly activates the parasympathetic system, had more benefits for women. Due to the small number of included studies that reported results stratified by gender, we were not able to conduct a gender-stratified meta-analysis. We did not see any significant impact of these continuous variables on results of depression and HRV. These results further emphasize that the effect of HRV-B in stress reduction might be population-specific. Age is also a well-known factor that influences HRV. A study (Aschbacher et al., 2024) found that age is associated with a significant decline in HRV-B amplitude. Another study (Yoo et al., 2022) found that younger adults showed a significant increase in spectral frequency power [F(1,95) = 45.33, *p* < 0.001], while older adults showed a marginally significant increase [F(1,51) = 3.85, *p* = 0.055]. However, our study did not show that age had a significant effect on HRV outcomes or other psychological outcomes in our meta-regression. This might be because there is a lack of older adults in the study population, as only 4 studies included adults above 50 years of age, while the average age of the rest of the study populations (k = 14) were much younger (32.98 years old).

With regards to categorical meta-regression results, practice time (over or under 20 min as it was approximately the average practice time of the studies), HRV-B method and interface had significant impacts on some intervention outcomes, mainly HRV and stress. For both stress and HRV, HRV-B interface—whether the interface was on the HRV-B device or a smartphone or other device, had an impact. For stress, studies that used smartphone apps or other software generally had worse intervention outcomes, while for studies with HRV as an outcome that had the interface directly on the HRV-B device had better outcomes. This could imply that having the screen on the HRV-B device increased the device’s ease of use and potentially made the intervention more effective. For HRV, studies that required daily practice times of 20 min or less had better outcomes. Though previous empirical studies have observed a dose–response relation (de Bruin et al., 2016; van der Zwan et al., 2015), the maximum practice time within these two studies was 20 min per day. Therefore, it is possible there is an inverted U-shape dose–response relationship with optimal benefit being 20 min/day. However, more study is required to establish the optimal daily practice duration. Finally, we found studies using the maximize resonance method to determine the individual breathing pace reported a greater increase in HRV than studies that used the stepped method. A previous systematic review mentioned the distinction between maximize resonance (they called it optimal resonance frequency) and the stepped method, however did not investigate the quantitative differences in how effective these methods are (Lalanza et al., 2023). Our results imply that this method is superior to the stepped method for increasing HRV (Table 2).

### Limitations

There are several limitations to the present study, including a small number of included studies, diversity of study populations, diversity of study designs, and risk of bias for the included studies. Although there were 18 studies included in the current systematic review, due to the transdiagnostic populations not all the studies examined the same symptoms. Therefore, the analyzable studies for each outcome were much smaller. Although the transdiagnostic nature of the populations allows for an understanding of the generalizability of the effects of HRV-B, the specificity of the effect in specific populations could not be examined, though separate meta-analyses of different population subgroups were conducted. Additionally, the diversity of study designs may also make the results difficult to interpret and compare, and only one of the studies included was low risk, the rest being medium to high risk. However, excluding non-randomized, outlier and high-risk studies did not change the results of the meta-analysis, implying that our results are relatively robust.

### Future Directions

This systematic review attempted to describe the methodology and effectiveness of prior empirical studies using remote HRV-B interventions in a transdiagnostic population. Future reviews could compare studies that only conducted interventions in the lab versus those that required home practice. Future empirical studies could also use the results of this review and meta-analysis regarding study protocol to help inform the design of their own protocol. Additionally, it may be useful to examine stress, anxiety, and sleep outcomes in particular as the results of this review were inconclusive for these outcomes due to not many papers published on the subject.

## Conclusions

HRV-B interventions may be moderately effective in treating depressive symptoms and increasing HRV across a variety of populations. The effect on stress might be population-specific and varied with intervention protocols. HRV-B intervention protocols differ in their approach and methodology, with HRV-B interface, method and practice time influencing some study outcomes. Specifically, the maximize resonance method in determining breathing pace and 20 min daily practice were found to be optimal for effect on HRV. These results provide some guidance for future studies. There are limited studies on specific outcomes and population. Therefore, further studies are needed to explore the effect of HRV-B interventions focusing on examining effects on specific outcomes such as stress, anxiety and sleep in specific populations.

## Supplementary Information

Below is the link to the electronic supplementary material.


Supplementary Material 1


## Data Availability

Data will be made available on request.
