# Supplementary material for: Efficacy and Methodology of Remote Heart Rate Variability Biofeedback Interventions for Mental Health: A Systematic Review and Meta-Analysis
Source: Appl Psychophysiol Biofeedback. 2025 Nov 27;51(3):441–52. doi: 10.1007/s10484-025-09750-w (PMC13525029; doi:10.1007/s10484-025-09750-w)
Supplement: Supplementary file 1 [file 10484_2025_9750_MOESM1_ESM.docx]

**Supplementary Figures:**

**S Figure 1:** Flow Chart of Intervention Procedure

**S Figure 2:** Forest Plots of Additional Outcomes

**S1a.** Sleep Forest Plot

**S1b.** Anxiety Forest Plot

*

n.s

p-val: 0.257

RE Model

**S Figure 3:** Leave One Out Sensitivity Analysis

**p-val**

**p-val**

**S Figure 3 Cont:** Leave One Out Sensitivity Analysis

**p-val**

**p-val**

**p-val**

**S Figure 4:** Risk of Bias Chart

Brinkmann et al, 2020

Burch et al, 2020

Cui et al, 2020

Cuneo et al, 2023

Eddie et al, 2014

Eddie et al, 2018

Kim, 2014

Kudo et al, 2014

Limmer et al, 2022

Schumann et al, 2022

Schumann et al, 2022

Tatschl et al, 2020

Vacher et al, 2023

Vagedes et al, 2019

van der Zwan et al, 2019

Wang et al, 2024

Winstead, 2019

Yen et al, 2023

**S Figure 4 Cont:** Risk of Bias Summary Chart

**S Figure 5:** Funnel Plots of Publication Bias

**S5b.** Stress Funnel Plot

Egger’s test: z = -2.56, p = 0.01, b = 1.04 (95% CI 0.12, 1.97)

**S5a.** Depression Funnel Plot

2: Eddie et al, 2018

5: Yen et al, 2023

8: Burch et al, 2020

9: van der Zwan et al, 2019

Egger’s test: z = 0.024, p = 0.981, b = -0.38 (95% CI -1.92, 1.17)

Egger’s test: z = -0.449, p = 0.654, b = -0.06 (95% CI -3.24, 3.12)

3: van der Zwan et al, 2019

Egger’s test: z = -2.178, p = 0.0294, b = 2.361 (95% CI 0.55, 4.17)

6: Kudo et al, 2014

9: Winstead, 2019

**S5c.** HRV Funnel Plot

Egger’s test: z = 1.795, p = 0.073, b = -1.03 (95% CI -2.65, 0.58)

**S5d.** Anxiety Funnel Plot

**S5e.** Sleep Funnel Plot

**Supplementary Tables**

|  | **k** | **Hedge's g** | **Upper** | **Lower** | **p** | **I² (%)** | **Q** | **pQ** | **Tau²** |
| --- | --- | --- | --- | --- | --- | --- | --- | --- | --- |
| **Depression *** | 10 | -0.410 | -0.049 | -0.772 | **0.026** | 72.623 | 32.608 | 0.000 | 0.238 |
| HRE * | 8 | -0.454 | -0.040 | -0.868 | **0.031** | 72.924 | 24.371 | 0.001 | 0.251 |
| OE ** | 9 | -0.524 | -0.209 | -0.840 | **0.001** | 61.077 | 21.219 | 0.007 | 0.136 |
| NRE * | 8 | -0.454 | -0.040 | -0.868 | **0.031** | 72.924 | 24.371 | 0.001 | 0.251 |
| **Stress** | 8 | -0.178 | 0.065 | -0.421 | 0.152 | 30.309 | 10.067 | 0.185 | 0.037 |
| HRE | 5 | -0.219 | 0.144 | -0.583 | 0.237 | 44.358 | 7.129 | 0.129 | 0.075 |
| OE | 8 | -0.178 | 0.065 | -0.421 | 0.152 | 30.309 | 10.067 | 0.185 | 0.037 |
| NRE | 6 | -0.167 | 0.123 | -0.458 | 0.259 | 33.125 | 7.363 | 0.195 | 0.043 |
| **HRV **** | 10 | 0.443 | 0.718 | 0.167 | **0.002** | 56.81 | 21.220 | 0.012 | 0.110 |
| HRE * | 7 | 0.312 | 0.566 | 0.058 | **0.016** | 31.020 | 7.934 | 0.243 | 0.036 |
| OE ** | 9 | 0.306 | 0.513 | 0.099 | **0.004** | 17.010 | 7.973 | 0.436 | 0.017 |
| NRE ** | 10 | 0.443 | 0.718 | 0.167 | **0.002** | 56.81 | 21.220 | 0.012 | 0.110 |
| **Sleep *** | 3 | -0.753 | -0.092 | -1.413 | **0.026** | 67.140 | 6.471 | 0.039 | 0.224 |
| HRE | 2 | -0.634 | 0.297 | -1.564 | 0.182 | 82.485 | 5.710 | 0.017 | 0.372 |
| OE * | 3 | -0.753 | -0.092 | -1.413 | **0.026** | 67.140 | 6.471 | 0.039 | 0.224 |
| NRE * | 3 | -0.753 | -0.092 | -1.413 | **0.026** | 67.140 | 6.471 | 0.039 | 0.224 |
| **Anxiety** | 3 | 0.304 | 0.829 | -0.222 | 0.257 | 60.075 | 4.869 | 0.088 | 0.129 |
| HRE | 1 | 0.390 | 0.957 | -0.177 | 0.177 | 0.000 | 0.000 | 1.000 | 0.000 |
| OE | 3 | 0.304 | 0.829 | -0.222 | 0.257 | 60.075 | 4.869 | 0.088 | 0.129 |
| NRE | 1 | 0.390 | 0.957 | -0.177 | 0.177 | 0.000 | 0.000 | 1.000 | 0.000 |

Negative values for psychometric outcomes (Depression and Stress) indicate greater improvement, positive values for HRV indicate greater increase. Abbreviations: k, number of studies; CI: confidence interval; HRE, High Risk Studies Excluded; OE, Outliers Excluded; NRE, Non-Randomized Studies Excluded. * <0.05 significance level; ** <0.01 significance level

95% CI

**S Table 1:** Meta-Analysis Results for Different Outcomes

| ***Outcome*** | ***k*** | ***Hedge's g*** | ***Upper*** | ***Lower*** | ***p*** | ***I²*** | ***Q*** | ***pQ*** | ***TAU*** |
| --- | --- | --- | --- | --- | --- | --- | --- | --- | --- |
| **Depression** |  |  |  |  |  |  |  |  |  |
| *Physical Condition* | 3 | 0.078 | 0.865 | -0.710 | 0.846 | 77.514 | 8.278 | 0.016 | 0.375 |
| Psychological Condition** | 6 | -0.583 | -0.192 | -0.974 | **0.003** | 64.269 | 14.806 | 0.011 | 0.146 |
| **Stress** |  |  |  |  |  |  |  |  |  |
| *Physical Condition* | 5 | -0.272 | 0.004 | -0.547 | 0.053 | 0.004 | 3.970 | 0.410 | 0.000 |
| Psychological Condition | 3 | -0.071 | 0.376 | -0.517 | 0.755 | 55.100 | 4.344 | 0.114 | 0.085 |
| **HRV** |  |  |  |  |  |  |  |  |  |
| *Physical Condition *** | 3 | 0.558 | 0.928 | 0.188 | **0.003** | 0.000 | 0.503 | 0.778 | 0.000 |
| Psychological Condition* | 7 | 0.411 | 0.786 | 0.036 | **0.032** | 69.554 | 19.749 | 0.003 | 0.175 |

**S Table 2:** Meta-Analysis Results by Population for Different Outcomes.

Negative values for psychometric outcomes (Depression and Stress) indicate greater improvement, positive values for HRV indicate greater increase. Abbreviations: k, number of studies; CI: confidence interval. * <0.05 significance level; ** <0.01 significance level

95% CI

**S Table 4:** Baseline Stress Meta-Regression

**S Table 3:** Diagnosis Subgroup Meta-Regression

**S2:** Continuous Meta-Regression Analyses for Different Outcomes

| ***Baseline Stress*** | **k** | **QM** | **QMp** | $\boldsymbol{\beta}$ | **SE** | **p-val** |
| --- | --- | --- | --- | --- | --- | --- |
| *Baseline Stress (Continuous)* | 8 | 0.583 | 0.445 |  |  |  |
| *Low-Moderate Stress* | 4 | 2.875 | 0.237 | -0.070 | 0.184 | 0.703 |
| *Moderate Stress* | 4 | 2.875 | 0.237 | -0.325 | 0.196 | 0.098 |

Abbreviations: k, number of studies; QM, omnibus test of moderators; QMp, p-value of QM; β, slope; SE, standard error. * <0.05 significance level; ** <0.01 significance level

|  | k | $\boldsymbol{\beta}$ | SE | Qm | p-val |
| --- | --- | --- | --- | --- | --- |
| Depression |  |  |  |  |  |
| *Cancer* | 1 | 0.746 | 0.672 | 8.889 | 0.267 |
| *Depression* | 1 | 0.783 | 0.686 | 8.889 | 0.253 |
| *Healthy* | 1 | 0.779 | 0.665 | 8.889 | 0.241 |
| *Migraine* | 1 | 0.679 | 0.670 | 8.889 | 0.311 |
| *Pregnant* | 1 | 0.162 | 0.639 | 8.889 | 0.800 |
| *PTSD* | 2 | 0.699 | 0.439 | 8.889 | 0.112 |
| *SMI* | 1 | 0.487 | 0.776 | 8.889 | 0.530 |
| *SUD* | 2 | 0.433 | 0.441 | 8.889 | 0.327 |
| Sleep |  |  |  |  |  |
| *Pregnant* | 2 | 0.551 | 0.468 | 4.701 | 0.238 |
| *SUD* | 1 | 1.105 | 0.607 | 4.701 | 0.069 |

| **Outcome** | **k** | **RR** | **Lower** | **Upper** | **p-val** |
| --- | --- | --- | --- | --- | --- |
| Depression ** | 10 | 1.324 | 1.106 | 1.586 | 0.004 |
| Sleep * | 3 | 1.785 | 1.360 | 2.343 | 0.019 |
| Anxiety ** | 3 | 1.681 | 1.301 | 2.173 | 0.004 |
| Stress * | 8 | 1.345 | 1.091 | 1.659 | 0.011 |
| HRV * | 10 | 1.236 | 1.007 | 1.519 | 0.041 |

Abbreviations: k, number of studies; QM, omnibus test of moderators; QMp, p-value of QM; β, slope; SE, standard error. * <0.05 significance level; ** <0.01 significance level

**S Table 5:** Relative Risk for Drop Out

95% CI

Abbreviations: k, number of studies; CI: confidence interval. * <0.05 significance level; ** <0.01 significance level
